# DNA Methylation at Birth Predicts Intellectual Functioning and Autism Features in Children with Fragile X Syndrome

**DOI:** 10.3390/ijms21207735

**Published:** 2020-10-19

**Authors:** Claudine M Kraan, Emma K Baker, Marta Arpone, Minh Bui, Ling Ling, Dinusha Gamage, Lesley Bretherton, Carolyn Rogers, Michael J Field, Tiffany L Wotton, David Francis, Matt F Hunter, Jonathan Cohen, David J Amor, David E Godler

**Affiliations:** 1 Diagnosis and Development, Murdoch Children’s Research Institute, Royal Children’s Hospital, Melbourne VIC 3052, Australia; claudine.kraan@mcri.edu.au (C.M.K.); emma.baker@mcri.edu.au (E.K.B.); marta.arpone@mcri.edu.au (M.A.); ling.ling@mcri.edu.au (L.L.); dinusha.gamage@mcri.edu.au (D.G.); lesley.bretherton@mcri.edu.au (L.B.); david.amor@mcri.edu.au (D.J.A.); 2 Department of Paediatrics, Faculty of Medicine, Dentistry and Health Sciences, University of Melbourne, Parkville VIC 3052, Australia; 3 School of Psychology and Public Health, La Trobe University, Bundoora VIC 3086, Australia; 4 Brain and Mind, Murdoch Children’s Research Institute, Royal Children’s Hospital, Parkville VIC 3052, Australia; 5 Centre for Epidemiology and Biostatistics, Melbourne School of Population and Global Health, University of Melbourne, Melbourne VIC 3052, Australia; mbui@unimelb.edu.au; 6 Genetics of Learning Disability Service (GOLD service), Hunter Genetics, Newcastle NSW 2298, Australia; Carolyn.Rogers@hnehealth.nsw.gov.au (C.R.); Mike.Field@health.nsw.gov.au (M.J.F.); 7 New South Wales Newborn Screening Program, Children’s Hospital at Westmead, Sydney NSW 2145, Australia; tiffany.wotton@health.nsw.gov.au; 8 Victorian Clinical Genetics Services, Murdoch Children’s Research Institute, Royal Children’s Hospital, Melbourne VIC 3052, Australia; david.francis@vcgs.org.au; 9 Monash Genetics, Monash Health, Clayton, VIC 3168, Australia; Matthew.Hunter@monashhealth.org; 10 Centre for Developmental Disability Health Victoria, Monash University, Doveton VIC 3177, Australia; jcohen@travelclinic.com.au; 11 Fragile X Alliance Inc., North Caulfield VIC 3161, Australia

**Keywords:** autism spectrum disorder (ASD), DNA methylation (DNAm), fragile X mental retardation 1 gene (*FMR1* gene), fragile X syndrome (FXS), intellectual disability (ID), newborn screening

## Abstract

Fragile X syndrome (FXS) is a leading single-gene cause of intellectual disability (ID) with autism features. This study analysed diagnostic and prognostic utility of the Fragile X-Related Epigenetic Element 2 DNA methylation (FREE2m) assessed by Methylation Specific-Quantitative Melt Analysis and the EpiTYPER system, in retrospectively retrieved newborn blood spots (NBS) and newly created dried blood spots (DBS) from 65 children with FXS (~2–17 years). A further 168 NBS from infants from the general population were used to establish control reference ranges, in both sexes. FREE2m analysis showed sensitivity and specificity approaching 100%. In FXS males, NBS FREE2m strongly correlated with intellectual functioning and autism features, however associations were not as strong for FXS females. Fragile X mental retardation 1 gene (*FMR1*) mRNA levels in blood were correlated with FREE2m in both NBS and DBS, for both sexes. In females, DNAm was significantly increased at birth with a decrease in childhood. The findings support the use of FREE2m analysis in newborns for screening, diagnostic and prognostic testing in FXS.

## 1. Introduction

DNA methylation (DNAm) is a key epigenetic modification associated with regulation of transcription that has been widely studied in human disease. It is influenced by environmental factors, such as diet [1] and smoking [2], as well as genetic factors [3], including pathogenic variants in genes regulating epigenetic machinery [4]. Aberrant DNAm has been associated with neurodevelopmental conditions that have complex multi-gene inheritance patterns (e.g., autism spectrum disorder (ASD)), sex specific changes observed as part of X-inactivation, as well as, locus specific changes in genetic architecture, as in trinucleotide disorders including fragile X syndrome (FXS) [5,6]. Importantly, DNAm analysis has been used for diagnostic testing in a number of neurodevelopmental disorders [4,6,7]. DNAm levels in neonatal samples including newborn blood spots (NBS), have also been found to correlate with adverse health outcomes (e.g., obesity) in offspring from mothers experiencing prenatal anxiety [8] and adiposity [9], and those exposed to drug use and other environmental toxins during pregnancy [2,10,11,12].

FXS, the focus of this study, is a common single gene cause of intellectual disability (ID) and co-morbid ASD (~1 in 4000 males and 1 in 5000 to 8000 females) [13]. FXS is caused by an expanded trinucleotide repeat (≥200 Cytosine-Guanine-Guanine (CGG)s) in the 5’ untranslated region of the fragile X mental retardation 1 gene (*FMR1)*, known as full mutation (FM). This FM usually causes silencing of *FMR1* through epigenetic modifications of the *FMR1* promoter including DNAm, and loss of *FMR1* protein (FMRP) essential for normal neurodevelopment [14,15]. In a significant proportion of individuals with FXS there is mosaicism with regards to CGG size and/or DNAm, such that a combination of active and silent *FMR1* alleles are present in the same individual, and can vary between tissues [16]. A common form of mosaicism is size mosaicism for premutation (PM: 55–199 CGG) and FM alleles (PM/FM).

The *FMR1* exon 1/intron 1 boundary, known as the Fragile X-Related Epigenetic Element 2 (FREE2) forms an extended portion of the *FMR1* promoter [6], and is sensitive to X-chromosome inactivation [17,18]. The extent of DNAm of this region has been shown to correlate with FMRP levels in blood and intellectual functioning in males and females affected with FXS [19,20,21,22]. FREE2 methylation (FREE2m) has been associated with presence of FM alleles in numerous tissue types, including: venous blood, saliva, lymphoblasts, chorionic villi [21,22,23], adult and newborn blood spots, epithelial cells, embryonic stem cells and primary neurons from post mortem brains [19,20,24,25,26,27], with some studies using a different name for the FREE2 region—for example *FMR1* “down-stream region” as in Esanov et al., [25]. Importantly, mRNA transcribed from the expanded CGG repeat has been shown to contribute to silencing of *FMR1*, by binding to the gene, with the highest affinity for binding shown for the FREE2 region, as compared to the rest of the promoter [28].

Moreover, for the larger *FMR1* promoter including the CpG island, DNAm has been reported to vary between tissues and cells (reflecting mosaicism) in its levels and location (down to single CpG resolution), between sexes and over time, as demonstrated by previous studies based on cross-sectional comparisons in FXS [16,26,29]. Notably though, little is known regarding the methylation profile throughout the lifespan in individuals with FXS, and a study of the longitudinal changes of FREE2m, from birth to adolescence, has not yet been undertaken. In addition, although the association between FREE2m in peripheral tissues, collected at time of assessment, and cognitive abilities in individuals with FXS has previously been explored, the relationships between FREE2m at birth, as analysed in retrieved NBS, and subsequent neurodevelopmental outcomes including autism features in children with FXS have not as yet been defined.

This study utilises retrospectively retrieved NBS (at birth) and dried blood spots (DBS; created at the time of assessment) collected from a large Australian cohort of children affected with FXS to determine: (i) sensitivity and specificity of FREE2m for FM alleles in NBS; (ii) whether the previously reported epigenotype–phenotype associations for FREE2m in older children [20,21], are conserved from birth; (iii) if and how FREE2m levels in NBS versus DBS are related to *FMR1* transcriptional changes, intellectual functioning and autism features throughout neurodevelopment in FXS in both sexes.

## 2. Results

**Figure 1:**
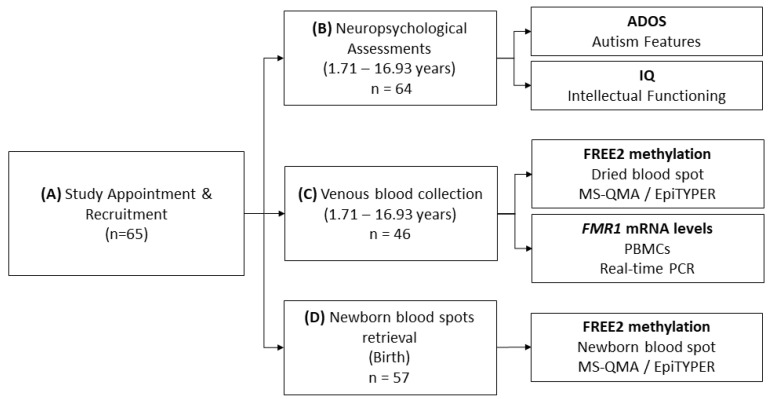
Methods and comparisons flowchart. (**A**) Sixty five Australian children with fragile X syndrome (FXS) aged 1.71 to 16.93 years old: (35.4% female) were recruited into the study and provided consent to: (**B**) undergo neuropsychological assessments (Autism Diagnostic Observation Schedule – Second Edition (ADOS-2) and Intelligence Quotient (IQ)); (**C**) provide blood to create dried blood spot (DBS) for Fragile X-Related Epigenetic Element 2 methylation (FREE2m) analyses and for isolation of peripheral blood mononuclear cells (PBMCs) for fragile X mental retardation 1 gene (*FMR1*) expression analyses; and (**D**) for retrieval of newborn blood spots (NBS) collected at birth for FREE2m analyses. The differences in numbers analysed for outcomes from (**A**) to (**D**) reflect differences due to proportion of participants that: (1) did not provide blood at recruitment, but did provide consent for NBS retrieval, where NBS samples could be located and retrieved; (2) did provide blood at recruitment and consent for NBS retrieval, where NBS samples could not be located and retrieved; (3) did not complete neuropsychological assessments and/or did not obtain valid scores. *FMR1* mRNA levels in PBMCs were analysed using the reverse transcription real-time PCR relative standard curve method [30]. FREE2m of NBS and DBS samples was analysed using Methylation Specific Quantitative Melt Analysis (MS-QMA) and the EpiTYPER system [19]. While both methods target the same locus consisting of 12 CpG sites, the EpiTYPER system is unable to analyse methylation of CpG’s 3, 4 and 5 as the cluster of fragments is too big in size (Daltons) to be captured by the mass-spectrum utilised by this system, as previously described [19,22]. For the remaining CpG sites the EpiTYPER system is able to provide CpG site-specific methylation. MS-QMA analysis provides a single aggregate measure of methylation across 11 out of 12 CpG sites (all but CpG 1).

The flowchart of procedures utilised in this study is shown in Figure 1. FXS cohort characteristics are presented in Table 1.

This study included five distinct analysis phases. Phase 1 determined sensitivity and specificity of FREE2m testing at birth (hereafter: time-point 1) to successfully differentiate NBS of FM only and PM/FM mosaic individuals from NBS of individuals with PM only and normal size (NS) alleles (Section 2.1). Phase 2 involved analyses of the relationships between FREE2m (i.e., time-point 1) in NBS and neuropsychological outcomes (determined at ~2 to 17 years of age: hereafter time-point 2) (Section 2.2). Phase 3 involved repeat of analyses of phase 2, but using FREE2m in DBS (i.e., time-point 2) as opposed to NBS (Section 2.3). Phase 4 involved analyses of the relationships between *FMR1* mRNA from PBMCs and both FREE2m in NBS (i.e., time-point 1) and DBS (time-point 2) (Section 2.4). Phase 5 examined change in FREE2m from time-point 1 to time-point 2 (Section 2.5). Phase 2-5 analyses were performed separately for children with only FM alleles, and for the combined FXS cohort of children with PM/FM mosaicism, and FM only alleles. Apart from phase 1, PM and NS groups were not included in the above analyses. Measurements of aggregate FREE2m in NBS was performed using two independent methods—Methylation Specific-Quantitative Melt Analysis (MS-QMA) and the EpiTYPER system in parallel on the same samples. These aggregate FREE2m measures from both methods were correlated in males (*n* = 32, r_s_ = 0.583, *p* = 0.001) and females (*n* = 20, r_s_ = 0.486, *p* = 0.048; Supplementary Tables S1 and S2).

### 2.1. Sensitivity and Specificity of FREE2m Analysis of NBS (Time-Point 1: Birth)

**Figure 2:**
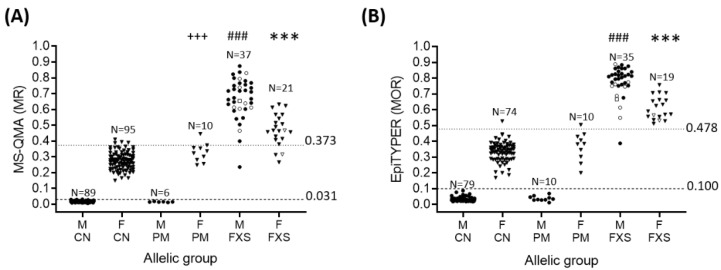
FREE2 aggregate methylation ratio (MR) determined using: (**A**) MS-QMA (CpG 2-12) in retrieved NBS samples stratified by gender and allelic group (males: 89 control (CN) newborns, and 6 PM and 37 FXS participants aged 1.89 to 16.93 years old at time of retrieval; females: 95 CN newborns, and 10 PM and 21 FXS participants aged 0.54 to 18.27 years old at time of retrieval); (**B**) FREE2 aggregate methylation output ratio (MOR) determined using the EpiTYPER system (mean CpG’s 1, 2, 6/7, 8/9 and 10-12 MOR) in the same cohort (males: 79 CN, and 10 PM and 35 FXS participants; females: 74 CN newborns, and 10 PM and 19 FXS participants. CN = control; F = female; FREE2 = fragile X-related epigenetic element 2; FXS: fragile X syndrome (FM and PM/FM); M = male; MS-QMA = methylation specific-quantitative melt analysis; PM = premutation. Circles represent males, triangles represent females, hollow symbols represent PM/FM mosaic individuals, and a hollow square represents the normal size (NS)/PM/FM mosaic individual (here NS represents alleles <44 CGGs). *** Significant difference between FXS males and PM and CN males (*p* < 0.001); ### significant difference between FXS females and PM and CN females (*p* <0.001); +++ significant difference between PM and CN females (*p* = 0.016). Dotted lines represent the optimal threshold for differentiating FXS participants from other groups, determined for males by the maximum control value and for females by the receiver operating characteristic curve. Significant *p* values are in bold.

Aggregate FREE2m analysis of NBS, using both MS-QMA and the EpiTYPER systems, differentiated all positive FXS males from control and PM cases with 100% sensitivity and specificity (Figure 2A,B). Using the aggregate NBS optimal threshold there was one PM female in the EpiTYPER “FXS range” and one in the MS-QMA “FXS range” (Figure 2). These two PM females were blood-related sisters. For females, MS-QMA had 85.71% sensitivity and 95.24% specificity, and the EpiTYPER system had 100% sensitivity and 98.78% specificity (Supplementary Table S3). For MS-QMA there were three potential false negatives and five potential false positives (one PM and four control females). For EpiTYPER, there was one potential false positive (PM female). One control female from the reference sample was an outlier on the EpiTYPER system and did not pass the quality control parameters as part of MS-QMA analysis. Thus, this female was excluded from sensitivity and specificity analyses.

### 2.2. Epigenotype-Phenotype Correlations using NBS FREE2m (Time-Point 1: Birth)

**Figure 3:**
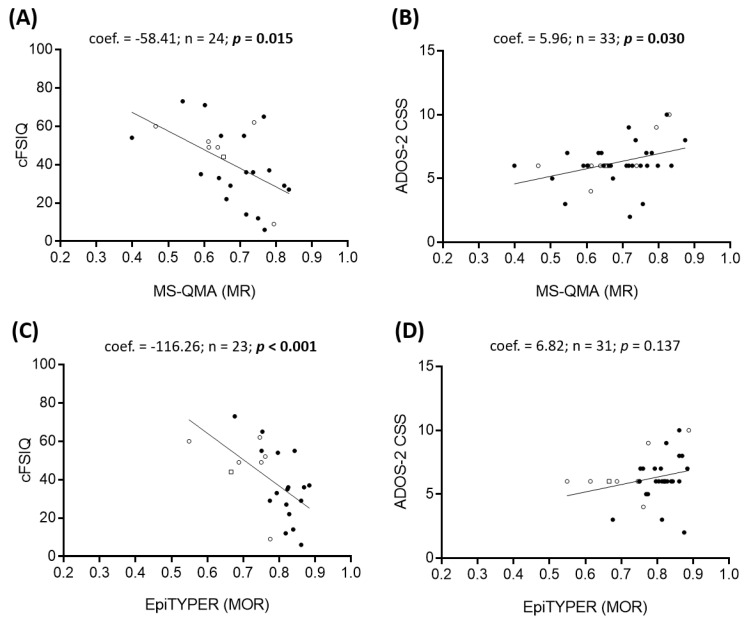
FREE2m assessed using MS-QMA and the EpiTYPER system in NBS samples from FXS males. FREE2 aggregate methylation ratio (MR) determined using MS-QMA (CpG 2-12) correlation with (**A**) corrected full scale IQ (cFSIQ) and (**B**) Autism Diagnostic Observation Schedule-Second Edition overall calibrated severity scores (ADOS-2 CSS); FREE2 aggregate methylation output ratio (MOR) determined using the EpiTYPER system correlation with (**C**) cFSIQ and (**D**) ADOS-2 CSS. FREE2 = fragile X-related epigenetic element 2; FXS: fragile X syndrome (FM only and PM/FM mosaic); MS-QMA = methylation specific-quantitative melt analysis. *Note*: Circles represent males, triangles represent females, hollow symbols represent PM/FM mosaic individuals, and a hollow square represents the normal size (NS)/PM/FM mosaic individual (here NS represents alleles <44 CGGs). Significant *p* values are in bold.

Aggregate FREE2m measured in NBS by MS-QMA in FXS males was significantly associated with corrected full scale intelligence quotient (cFSIQ) (Figure 3), corrected verbal intelligence quotient (cVIQ) and corrected performance intelligence quotient (cPIQ) (Supplementary Table S4) and the Autism Diagnostic Observation Schedule--Second Edition (ADOS-2) calibrated severity score (CSS) (Figure 3; Supplementary Table S5). Similarly, aggregate FREE2m measured in NBS by the EpiTYPER system in FXS males was significantly associated with cFSIQ (Figure 3) and both cVIQ and cPIQ (Supplementary Table S4). In FXS females there were no statistically significant associations between aggregate FREE2m in NBS (using either platform) and corrected IQ scores or autism features (Supplementary Tables S4 and S5). However, the strength of the association between aggregate FREE2m measured by the EpiTYPER system and cVIQ (Supplementary Table S4) was similar to that reported for MS-QMA and cVIQ in the male sample. The smaller sample size and greater variability in females (reflected in standard error) likely reduced power to detect a statistically significant association.

**Figure 4:**
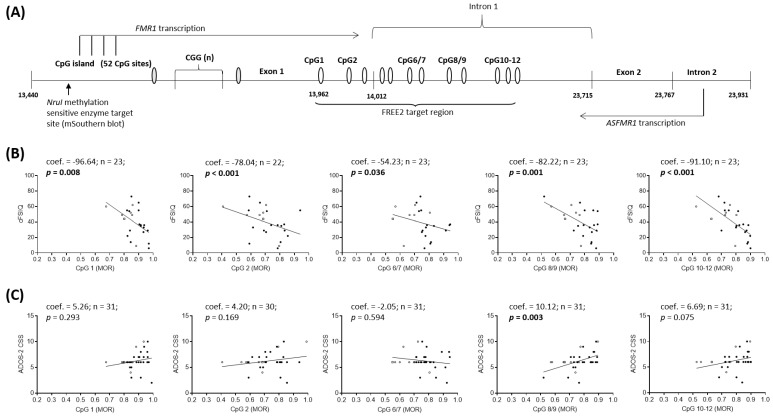
Correlations in FXS males between FREE2m levels of individual CpG sites in NBS samples using the EpiTYPER system, and cFSIQ and ADOS-2 CSS results determined at time of recruitment. (**A**) Organisation of the Xq27.3 sequence encompassing specific FREE2 CpG sites (GenBank L29074 L38501) targeted by the EpiTYPER system. Correlations between FREE2m levels and: (**B**) cFSIQ in 23 FXS males aged 3.32 to 16.93 years old; and (**C**) ADOS-2 CSS in 31 FXS males aged 1.89 to 16.93 years old. Note: Hollow circles represent PM/FM mosaic cases and a hollow square represents the normal size (NS)/PM/FM male. ADOS-2 = Autism Diagnostic Observation Schedule-Second Edition; CSS = Calibrated Severity Score; FREE2 = Fragile X-Related Epigenetic Element 2; MS-QMA = methylation specific-quantitative melt analysis. Significant *p* values are highlighted in bold.

In line with the aggregate measures, CpG site specific DNAm was significantly associated with all three corrected intellectual functioning scores (cFSIQ, cVIQ and cPIQ) in FXS males (Supplementary Table S4). DNAm of CpG’s 1, 2, 8/9 and 10-12 showed the strongest associations (Figure 4; Supplementary Table S4). In FXS males both the ADOS-2 CSS and social affect (SA) CSS, significantly associated with DNAm of CpG 8/9 (Figure 4; Supplementary Table S5). In FXS females there were no statistically significant associations detected between CpG site-specific DNAm and IQ or ADOS-2 scores (Supplementary Tables S4 and S5). However, the strength of the association between CpG 2 and cFSIQ and cVIQ and CpG 6/7 with cFSIQ and cVIQ were similar to those found in the male sample (Supplementary Table S4). A similar strength of association was also observed in the female group between DNAm and ADOS-2 CSS and SA CSS as shown in the male group (Supplementary Table S5). The results for both males and females were largely unchanged when the children with PM/FM mosaicism were excluded from the analyses (Supplementary Tables S6 and S7).

The analysis was also performed with a subsample of children with all data available (i.e., both NBS and DBS results). This sub-sample included 12 females (mean age = 7.43 years; 83.33% FM only) and 14 males (mean age = 7.55 years; 78.57% FM only). Results were similar in this subsample of males and females for associations between FREE2m in NBS and corrected IQ and ADOS-2 scores (Supplementary Tables S8 and S9). However, CpG 8/9 DNAm in NBS was no longer significantly associated with any corrected IQ or ADOS-2 scores in males and only aggregate FREE2m by MS-QMA was significantly associated cVIQ scores. In contrast, CpG 2 and CpG 8/9 were significantly associated with cVIQ in females in this subsample (Supplementary Table S8).

### 2.3. Epigenotype-Phenotype Correlations using DBS FREE2m (Time-Point 2: Childhood)

In FXS males, DNAm of CpG 2 was associated with cPIQ (Supplementary Table S10) and DNAm of CpG 6/7 was associated with restricted and repetitive behaviours (RRB) CSS (Supplementary Table S11), but were no longer significant after adjustment for multiple testing. There were no other statistically significant associations between DNAm and corrected IQ scores or ADOS-2 variables, in either the male and female groups (Supplementary Tables S10 and S11). Similar to the epigenotype-phenotype analyses in NBS, the results were largely unchanged when only children with FM were included in the analyses (Supplementary Tables S12 and S13).

The analyses with the subsample of only those children with complete data were similar to the findings for the complete cohort. Aggregate DNAm in DBS analysed by the EpiTYPER system was significantly associated with both cVIQ and cPIQ in FXS males (Supplementary Table S14). For the site-specific analyses, only cPIQ remained significantly associated with DNAm of CpG 2 and CpG 10-12 in FXS males after adjusting for multiple testing (Supplementary Table S14). None of the DBS FREE2m variables were significantly associated with autism features in males and females with FXS with complete data (Supplementary Table S15).

### 2.4. Correlation between FMR1 mRNA and FREE2m Using NBS (at Birth) and DBS (during Childhood)

In FXS males and females, aggregate EpiTYPER and site-specific DNAm levels in both NBS and DBS correlated significantly with *FMR1* mRNA levels in blood (Supplementary Table S16). In FXS males, NBS CpG 8/9 and DBS CpG 2 correlated with *FMR1* mRNA levels. However, these correlations were lost after exclusion of PM/FM individuals (Supplementary Table S17). In FXS females, correlations were strong, found consistently across all CpG units, except CpG 2, and were not affected by inclusion of one female with PM/FM mosaicism. The correlation between *FMR1* mRNA and DNAm in DBS was stronger than the correlation observed with DNAm in NBS for FXS females for all sites except for CpG 10-12, which was comparable between DNAm in NBS and DBS (Supplementary Table S16). The results of these analyses were similar when only those with complete data were included (Supplementary Table S18).

### 2.5. Longitudinal Changes in FREE2m from Birth to Childhood

Longitudinal changes in aggregate and CpG site-specific DNAm were analysed at group level using the Wilcoxon signed-ranks test, to test for a significant difference from zero for annual change ((follow up—baseline)/age)). In FXS males, group annual DNAm change from birth to time of assessment (i.e., 1.89 to 16.93 years old) was not significantly different. By contrast, in FXS females, there was a significant group decrease in annual DNAm change from birth to time of assessment (i.e., 1.71 to 13.06 years old) for most DNAm measures (excluding MS-QMA; Table 2).

## 3. Discussion

This is the first study to demonstrate associations between DNAm at birth, from retrospectively retrieved NBS, and the clinical phenotype during childhood in FXS males, and to a lesser extent in females. Importantly, these associations were observed using two independent platforms, MS-QMA and the EpiTYPER system. Specifically, in males with FXS, increased FREE2m in NBS was associated with lower intellectual functioning and greater autism features during childhood (1.78 to 16 years). Interestingly, despite DBS being created from venous blood collected at the time of the clinical assessment, there were fewer associations between FREE2m in DBS and the clinical phenotype compared to NBS. Artefactual issues such as how blood was collected or time since collection could have impacted results. These findings also suggest that environmental factors may play a critical role in epigenotype–phenotype associations during child development in FXS.

Additionally, FREE2m was shown to identify cases with FM alleles in both males and females with sensitivity and specificity approaching 100%. These findings demonstrate that FREE2m in newborns can effectively screen for FXS in male and female infants and predict neurodevelopmental outcomes in FXS males, and to a lesser extent in FXS females. Using FXS as a model, the findings also highlight that comparisons between FREE2m in retrospectively retrieved NBS from newborns and DBS during childhood, provide a unique, convenient, and cost-effective avenue to study longitudinal changes in DNAm more broadly in paediatric disease.

### 3.1. Epigenotype-Phenotype Associations

FREE2m levels at birth were associated with the clinical phenotype at the time of assessment including intellectual functioning and autism features. The associations between FREE2m at birth and intellectual functioning in FXS males found in this study support previous findings of an association between cFSIQ and FREE2m in DNA derived from buccal epithelial cells in FXS males aged 3 to 17 years old [20]. The current study for the first time shows that these associations in blood are conserved from birth, as demonstrated in retrospectively retrieved NBS samples. The study also demonstrates association between FREE2m at birth and autism features, specifically social communication deficits, in FXS males. This adds weight to the clinical importance of the present relationships, as FXS with autism is associated with poorer outcomes [31,32]. These findings also provide support for the prognostic utility of DNAm testing that targets the FREE2 region in FXS cohorts.

Moreover, when only those children with complete data (i.e., both NBS and DBS results) were included in the analyses, findings were consistent with previous research conducted in FXS females with a broader age range (up to 35 years old), in which DNAm of CpG sites within the intron was correlated with verbal skills [21]. Although there was evidence for these same associations in the present investigation of female children with FXS, independent validation using larger sample sizes is needed to further replicate and confirm these findings.

### 3.2. Screening and Diagnostic Potential

The FREE2m positive and negative DNAm reference ranges provided in this study are consistent with previous smaller studies of MS-QMA and EpiTYPER platforms in de-identified NBS in a different patient cohort [19] and cross-sectional studies of FREE2m in different tissues [18,19,21,22]. Together these findings support the use of FREE2m analysis on NBS material remaining after completion of standard neonatal screening to provide molecular diagnosis of FXS, avoiding collection of new biological samples which can be difficult for severely affected children. The high sensitivity and specificity for cases with FM alleles also supports the use of MS-QMA as a first line test (due to the low reagent cost) in males and females for large scale newborn screening studies. Importantly, the method used is in line with the minimum material requirements for such programs (3mm punch per newborn) [19].

We demonstrate that when both MS-QMA and EpiTYPER are used to analyse FREE2m in NBS, all PM males and most PM females show methylation ratio values below the optimal “FXS-positive” threshold (highlighted by dotted lines in Figure 2). This is important in a newborn screening context because PM alleles have been linked to increased risk of developing adult onset neurodegenerative conditions with no cure and incomplete penetrance [33]. Based on this finding, we propose a two-step protocol of MS-QMA followed by EpiTYPER as a potential method specific for FM alleles in both sexes, that does not identify PM alleles in newborns, because methylation of PM alleles largely overlaps with methylation of NS alleles. If verified in larger independent cohorts, this method could address concerns around detecting a high number of PM alleles in potential FXS newborn screening programs.

The study results are consistent with the findings of Hensel and colleagues [34], in an older independent cohort of males and females identified through diagnostic testing and in a different tissue. This previous study demonstrated that FREE2m levels in buccal epithelial cells determined by MS-QMA had diagnostic sensitivity and specificity approaching 100% in both sexes.

Interestingly, the two PM females that could be considered as “false positives” (one by MS-QMA and one by EpiTYPER) in this study were sisters, with the PM being paternally inherited. The elevated DNAm in these females may be due to the presence of a cryptic FM expansion in a small proportion of cells, that may have been missed by standard testing, the occurrence of which was recently reported in an independent cohort [34]. Expansion from a PM to FM has been considered to occur exclusively through vertical transmission from females. Nonetheless, two case reports of PM/FM females with paternal inheritance have been described [35,36]. Unfortunately, due to a lack of sufficient DNA quantity, no further CGG sizing analyses could be performed to investigate the possibility of mosaic FM alleles in the two females in this study, that were not detected by standard of care FXS testing.

### 3.3. Sex Specific Differences in DNAm Longitudinal Trajectories in Blood

In FXS males, longitudinal analysis at group level did not reveal significant differences in DNAm at the FREE2 region between time-points one (birth) and two (childhood). However, in DBS collected at the time of assessment of FXS males (childhood), there were fewer significant correlations between DNAm and the phenotype as compared to NBS, despite similar correlations with *FMR1* mRNA in NBS and DBS samples. This discrepancy between NBS and DBS epigenotype–phenotype associations was also observed in those children who had complete data. This phenomenon could be explained by inter-individual heterogeneity in DNAm over time in the male group, possibly related to stochastic variability and changes due to developmental and environmental factors (e.g., family and home environment, behavioural interventions, medication use, etc.). Moreover, DNAm change has been indicated to be highly dynamic in the first 5 years of life [37,38], the period when children are most likely to undergo diagnostic testing.

In FXS females, there was significantly increased FREE2m (at aggregate and CpG site-specific level) at birth, which then decreased in childhood. This female specific decrease in DNAm over time extends the findings from previous cross-sectional studies which have shown in adult FXS females, that increased age is negatively correlated with FREE2m [26] and CpG island methylation [39,40,41]. These are the first longitudinal findings to support such a relationship, and the first to demonstrate that the relationship holds in younger FXS females starting from birth. Since all FXS females are in essence mosaic for cells that have pathogenic FM alleles either on the active or inactive X chromosome, those females that had the most rapid selection against cells in blood that have FM on the active X (represented by the greatest decrease in annual DNAm change from birth) may also be individuals who have a higher proportion of these cells with “less functional *FMR1*/FMRP” in the brain at birth.

Together these data suggest that stochastic changes over time at the *FMR1* promotor due to environmental, clonal selection related to RNA toxicity of unmethylated FM alleles or other factors could impact the diagnostic and prognostic utility of DNAm levels when analysed in peripheral tissues, such as blood which is usually tested. This could have broad implications for diagnostic and prognostic testing for both FXS and other neurodevelopmental disorders currently diagnosed through targeted DNAm analysis of peripheral tissues, and for measuring the degree of X-chromosome inactivation skewing in X-linked conditions using DNAm based analyses.

### 3.4. Limitations

Limitations of this study include: (i) one time-point for phenotype data; and (ii) potential use of a study cohort that may not reflect the wider FXS population, as our male cohort was low functioning (ascertainment bias). Sample characteristics (i.e., age) and use of different neuropsychological testing batteries may also explain differences from the previous cross-sectional studies. Moreover, despite the close correlations between the two platforms used in this study (aggregate and site-specific), there are important distinctions. Specifically, although MS-QMA and the EpiTYPER system analyse the same FREE2 region, within that region, CpG 3-5 DNAm cannot be detected by the EpiTYPER system. This cluster of fragments is too big in size (Daltons) to be captured by the mass-spectrum (Figure 4A). In contrast, the aggregate measure of FREE2m from MS-QMA incorporates DNAm of the CpG 3-5 cluster, as MS-QMA does not employ mass-spectrometry. Conversely, MS-QMA does not capture methylation of CpG 1 which is included in aggregate DNAm analysis using the EpiTYPER system. These differences between platforms may explain differences (at site-specific and aggregate levels) in the sensitivity and specificity of the two different FREE2m platforms for FXS individuals and the associations with phenotypic variables reported in this study. A prospective longitudinal study (e.g., birth cohort design), with phenotypic assessments and collection of biological samples at equal and consistently spaced time points would address some of the limitations present in the current study.

## 4. Materials and Methods

### 4.1. Participants

The FXS cohort included 65 Australian children aged 1.71 to 16.93 years old: (35.4% female) (Figure 1). Within this group, two (9%) females and 11 (26%) males were PM/FM mosaic, 21 (91%) females and 31 (74%) males were FM only. One child who had a formal diagnosis of PM/FM mosaicism was found to also have a normal size allele (NS/PM/FM) when further testing was undertaken using AmplideX PCR, a commercial CGG sizing assay (Asuragen, Austin, TX, USA). Allelic class represented the diagnosis provided to the family on the formal diagnostic report (i.e., PCR followed by Southern Blot and/or AmplideX PCR sizing). Participants were recruited nationally through: (i) Victorian Clinical Genetics Services (VCGS), Murdoch Children’s Research Institute and Genetics of Learning Disability Service (GOLD), Hunter Genetics; (ii) referring practitioners; and (iii) family support groups. Twenty-four of the FXS males included in the current study were included in a cross-sectional study examining the associations between DNAm in buccal epithelial cells and intellectual functioning [20]. None of the females included in this study have been included in previous epigenotype–phenotype studies. Study exclusion criteria were based on the presence of significant medical or neurological condition(s) including: stroke, malignancies, severe head trauma, congenital heart disease, liver or renal failure, inadequate control of seizures, history of prolonged bleeding or abnormal platelet count or if they were pregnant. All participants’ parents or guardians, and those participants who were cognitively able, signed an informed consent or had a legally acceptable representative provide consent. All study procedures were approved by the Royal Children’s Hospital Human Research Ethics Committee (Single Site reference number: HREC #33066; Multi Site reference number HREC/13/RCHM/24) on 24 May 2013.

NBS reference ranges for: (i) NS (CGG<44) alleles were established on 184 de-identified samples from infants where CGG size was confirmed in previous studies [19], (ii) PM alleles were established on retrospectively retrieved NBS from 22 children (~55% female) with a PM allele (male: 57–130 CGG; female 57-165 CGG), aged 0.54 to 18.27 years old at time of participation in the study.

Allele sizes for NS controls were confirmed to be in the normal range (CGG < 44) using PCR methodologies and denaturing capillary electrophoresis. De-identified control NBS were collected from individuals who participated in a fragile X feasibility study by the New South Wales (NSW) Newborn Screening Programme and Department of Molecular Genetics at the Children’s Hospital, Westmead, Australia [42].

### 4.2. Molecular Testing

Control NBS were accessed through an Australian Newborn Screening Program. NBS from study FXS and comparison PM participants were retrieved from newborn screening laboratories in four Australian states (VIC, NSW, WA and SA). For FXS participants DBS samples were also created at the time of participation in the study during childhood, when venous blood could be collected. Here 50 microlitres of blood was dropped onto the same Guthrie card filter paper as used for NBS sample collection, with processing performed as previously described [18,19].

FREE2m was analysed using two independent methods, MS-QMA and the EpiTYPER system. The EpiTYPER system provided methylation of 5 CpG units—encompassing 9 CpG sites (CpG 1, CpG 2, CpG 6/7, CpG 8/9, CpG 10–12) within the FREE2 region, and an aggregate measure representing mean methylation across these units [19]. MS-QMA provided one aggregate measure of DNAm of the same locus. Both MS-QMA and EpiTYPER analyses were performed on bisulfite converted DNA from a single 3mm DBS punch, as previously described [19]. Methylation analysis for each DNA sample was performed in duplicate bi-sulphate conversions, with each conversion analysed twice using the EpiTYPER system and MS-QMA. The mean of the 4 methylation ratio measurements per sample was used as a summary measure for each CpG unit and/or aggregate DNAm measure analysed. The technical variability between these replicates did not exceed 2%, for MS-QMA as described in Figure 1 of Inaba et al. [19] and 5% for the EpiTYPER system, as described in Cornish et al. [43] (Figure e-1). There was a slight difference between the number of NBS and DBS samples analysed between MS-QMA and the EpiTYPER system, because MS-QMA analysis was performed first, and for some samples where only one 3 mm blood spot was available, there was insufficient DNA lysate remaining for analysis using the EpiTYPER system.

Peripheral blood mononuclear cells (PBMC) were isolated from 5 millilitres of venous blood, for RNA extraction for gene expression analyses using the reverse transcription real-time PCR on a ViiA 7 Real-Time PCR System (Thermo Fisher Scientific, Carlsbad, CA, USA). The relative standard curve method was utilised for *FMR1* 5′ and 3′ mRNA quantification normalised to mRNA of 2 internal control genes (*EIF4A2* and *SDHA*) [30].

### 4.3. Neuropsychological Testing

Neuropsychological testing was performed on FXS participants by an assessor blinded to the allelic classification. Intellectual functioning was assessed with either the Wechsler Preschool and Primary Scale of Intelligence—Third Australian Standardised Edition (WPPSI-III Australian) [44] or the Wechsler Intelligence Scale for Children—Fourth Australian Standardised Edition (WISC-IV Australian) [45] for children aged 3 years and above. In the current study, IQ scores were represented by “corrected IQ” scores (e.g., cFSIQ) as previously described [20]). This method allows extrapolation below the floor level and has previously been shown to be strongly correlated with DNAm in buccal epithelial cells in a sample of males with FXS [20]. The Autism Diagnostic Observation Schedule—Second Edition (ADOS-2) [46] was administered to all but one FXS participant, with overall calibrated severity scores (CSS), social affect (SA) CSS and restricted and repetitive behaviours (RRB) CSS scores selected for analyses.

### 4.4. Statistical Analyses

Summary statistics were presented by mean and standard deviation (SD) when the data were normally distributed, and comparisons between sex were performed using two-sample t-tests. For data with a skewed distribution, median and interquartile range, and the non-parametric Mann–Whitney test, were used. For all analyses, aggregate MS-QMA and EpiTYPER results were analysed, followed by CpG site-specific analyses.

A non-parametric receiver operating characteristic curve (ROC) was used as diagnostic test evaluation to compare performance of FREE2m (aggregate) results for selecting FXS versus PMs and controls. Optimal threshold from the ROC was determined by the Youden method, and this threshold was used to dichotomise the data into above and below groups to estimate sensitivity, specificity and area under the curve (AUC).

Associations between each DNAm predictor and each outcome variable (i.e., intellectual functioning and autism features) were assessed using linear regression, estimated by the least square method if outliers were not present, otherwise robust regression was used. These associations were adjusted for age and psychotropic medication use, if significantly correlated with the outcome variable. For the epigenotype–phenotype analyses, 7 FXS participants (2 females and 5 males) were randomly excluded due to being related to another participant of the same sex (i.e., twin or sibling). Associations between each FREE2m variable and *FMR1* mRNA levels were examined using Spearman’s rank correlation (r_s_). Analyses were run separately in NBS and DBS, for males and females with FM only alleles, as well as for combined FXS cohort of FM only and PM/FM mosaic participants. Not all children provided a venous blood sample at the time of assessment for creation of a DBS and in some instances NBS could not be retrieved. Thus, a sub-analysis was conducted including only those children with a complete dataset (NBS, DBS, IQ and ADOS) to reduce the possibility of participant characteristics (epigenotype and phenotype) biasing results and differences between time points.

Annual change, defined as the difference in FREE2m between follow-up and baseline (newborn) divided by age, was used to study longitudinal methylation change. This was selected due to the different lengths of time from birth to DBS time-points across participants. One-sample t-test was used to determine whether the annual change was different from zero, or Wilcoxon signed-ranks test if data were not normally distributed.

False discovery rate (FDR) was used to adjust for multiple testing for analyses utilising CpG site-specific measures. FDR was not applied to aggregate measures of FREE2m. All analyses were conducted using STATA version 15.0 (www.stata.com) and *p*-values were 2-sided with significance at <0.05.

## 5. Conclusions

This study for the first time demonstrates that FREE2m analysis using both MS-QMA and the EpiTYPER system is a feasible approach for newborn screening, with sensitivity and specificity approaching 100% to detect abnormally methylated FM alleles in both sexes. The key novel findings were that FREE2m analysis in male, and to a lesser extent in female, infants was predictive of intellectual functioning and autism features when they become children affected with FXS. This has the potential to open new avenues for detection of FXS FM alleles in newborn blood spots in both sexes and for prognostic testing in newborns and children as they develop.

## 6. Patents

David Eugeny Godler is named as an inventor on patent applications (PCT/AU2010/000169, PCT/AU2011/001024 and PCT/AU2014/000044) related to the technology described in this article. 

## Figures and Tables

**Table 1:** Characteristics of the FXS cohort.

	FXS (FM + PM/FM)					FM only				
	Male (*n* = 42)		Female (*n* = 23)			Male (*n* = 31)		Female (*n* = 21)		
	N	Mean ± SD	N	Mean ± SD	*p*	N	Mean ± SD	N	Mean ± SD	*p*
Age at assessment ^a^	42	5.09 ± 4.60	23	5.02 ± 3.63	0.8103	31	5.17 ± 4.80	21	5.20 ± 4.12	0.8447
cFSIQ	33	41.1 ± 18.4	18	61.3 ± 17.2	**0.0004+**	23	38.2 ± 19.3	16	58.6 ± 15.8	**0.0013+**
cPIQ	33	47.3 ± 16.0	18	61.9 ± 16.0	**0.0031+**	23	45.6 ± 17.2	16	60.3 ± 15.9	**0.0102+**
cVIQ	33	51.5 ± 14.8	18	68.3 ± 16.2	**0.0005+**	23	49.9 ± 15.4	16	65.8 ± 15.0	**0.0028+**
ADOS-2 CSS ^a^	42	6.00 ± 1.00	22	4.00 ± 3.00	**0.0002+**	31	6.00 ± 1.00	20	4.00 ± 2.75	**0.0012+**
SA CSS ^a^	42	6.00 ± 3.00	22	4.00 ± 3.00	**0.0094+**	31	6.00 ± 3.00	20	4.00 ± 2.75	**0.0226+**
RRB CSS ^a^	42	8.00 ± 3.00	22	7.00 ± 2.25	**0.0012+**	31	8.00 ± 3.00	20	7.00 ± 2.00	**0.0207+**
Gestational age ^a^	41	39.0 ± 3.00	23	39.0 ± 2.00	0.5256	30	38.0 ± 3.00	21	39.0± 2.00	0.2914
Medication use ^b^	42	38.1%	23	26.1%	0.4157	31	35.5%	21	28.6%	0.7650
Ethnicity ^b^	42		23		0.8537	31		21		0.8305
White	32	76.2%	18	78.3%		22	71.0%	16	76.2%	
Other	9	21.4%	4	17.4%		8	25.8%	4	19.0%	
Not disclosed	1	2.4%	1	4.3%		1	3.2%	1	4.8%	

The male FXS group comprised 31 FM and 11 individuals mosaic for PM/FM alleles. The female FXS group comprised 21 FM and 2 individuals mosaic for PM/FM alleles. A two-sample t-test was used to test the difference in mean (± SD) between sex, otherwise a nonparametric ^a^ Mann–Whitney test was used to test the difference in medians (± interquartile). For binary data (medication use and ethnicity), a ^b^ Chi square test was used to test the difference in proportions between two groups. For females the ‘other’ ethnicity included: Iranian (*n* = 1), Native Australian (*n* = 1), mixed ethnicity (*n* = 2); for males the “other” ethnicity included: Iranian (*n* = 2), Vietnamese (*n* = 1), Sri Lankan (*n* = 1), mixed ethnicity (*n* = 5). ADOS-2 = Autism Diagnostic Observation Schedule--Second Edition; CSS = calibrated severity score; cFSIQ = corrected full scale intelligence quotient; cPIQ = corrected performance intelligence quotient; cVIQ = corrected verbal intelligence quotient; FM = Full mutation allele (defined as Cytosine-Guanine-Guanine (CGG) ≥ 200 repeats); PM = premutation alleles (CGG 55-199 repeats); RRB = restricted and repetitive behaviours; SA = social affect; +*p*-value (*p*) in bold remained <0.05 after adjustment for multiple testing.

**Table 2:** FREE2m annual change in FXS males and females.

		Male				Female		
	N	Mean	SD	*p*	N	Mean	SD	*p*
MS-QMA *	20	0.56 ^a^	2.43	0.067	15	–0.50	1.99	0.351
EpiTYPER #	18	–0.30 ^a^	1.22	0.286	15	–0.76 ^a^	1.58	**0.008**
CpG 1	18	–0.72	3.02	0.328	15	–0.38	2.67	0.585
CpG 2	16	–0.45 ^a^	2.01	0.326	13	–1.06	1.13	**0.006+**
CpG 6/7	17	–0.40 ^a^	1.79	0.332	12	–1.06	1.64	0.047
CpG 8/9	17	–0.12	3.91	0.897	14	–1.56	2.11	**0.016+**
CpG 10-12	18	–0.50	1.89	0.282	14	–1.13 ^a^	1.73	**0.001+**

Annual FREE2m change ((follow up–baseline)/age)) was tested by the Wilcoxon signed-ranks test for a significant difference from zero. All summarised statistics are multiplied by 100. ^a^ Variables were not normally distributed, therefore median and interquartile range were used. * Aggregate of CpG’s 2-12; # aggregate computed as mean DNAm at CpG sites 1, 2, 6/7, 8/9 and 10–12). Repeat of these analyses excluding the PM/FM mosaic individuals did not change the results (i.e., no significant change for males; significant decrease over time in females for CpG 2 (*p* = 0.019), CpG 8/9 (*p* = 0.016), CpG 10-12 (*p* = 0.002) and the EpiTYPER aggregate value (*p* = 0.019). + *p*-value in bold (*p*) remained < 0.05 after adjustment for multiple testing of CpG 1 to CpG 10-12.

## Supplementary Materials

Supplementary materials can be found at https://www.mdpi.com/1422-0067/21/20/7735/s1.

## Abbreviations

ADOS-2	Autism Diagnostic Observation Schedule-Second Edition
ASD	Autism Spectrum Disorder
AUC	Area under the curve
cFSIQ	Corrected Full Scale IQ
CGG	Cytosine-Guanine-Guanine
cVIQ	Corrected Verbal IQ
CSS	Calibrated Severity Score
cPIQ	Corrected Performance IQ
DBS	Dried blood spots
DNAm	DNA methylation
FDR	False Discovery Rate
FM	Full mutation
FMRP	Fragile X Mental Retardation Protein
FREE2	Fragile X Related Epigenetic Element 2
FREE2m	Fragile X Related Epigenetic Element 2 methylation
FXS	Fragile X syndrome
ID	Intellectual Disability
IQ	Intelligence Quotient
MS-QMA	Methylation Specific-Quantitative Melt Analysis
NBS	Newborn blood spots
NS	Normal size
PBMC	Peripheral Blood Mononuclear Cells
PCR	Polyamerase Chain Reaction
PM	Premutation
PM/FM	Premutation/Full mutation
ROC	Receiver operating characteristic curve
RRB	Repetitive and Restricted Behaviours
SA	Social Affect
VCGS	Victorian Clinical Genetic Services
WPPSI-III	Wechsler Preschool and Primary Scale of Intelligence—Third edition
WISC-IV	Wechsler Intelligence Scale for Children—Fourth edition
