# Supplementary material for: DNA Methylation at Birth Predicts Intellectual Functioning and Autism Features in Children with Fragile X Syndrome
Source: Int J Mol Sci. 2020 Oct 19;21(20):7735. doi: 10.3390/ijms21207735 (PMC7589848; doi:10.3390/ijms21207735)
Supplement: Supplementary file 1 [file ijms-21-07735-s001.pdf]

Table S1: Correlations between MS-QMA and EpiTYPER in NBS and DBS samples in FXS males.

Table S2: Correlations between MS-QMA and EpiTYPER in NBS and DBS samples in FXS females.

Table S3: Sensitivity and specificity of NBS FREE2m for FXS versus PM and control females.

Table S4: Association between each corrected intellectual functioning score and each NBS FREE2m in FXS males and females.

Table S5: Association between each autism features (ADOS-2 scores) and each NBS FREE2m in FXS males and females.

Table S6: Association between corrected intellectual functioning scores and NBS FREE2m in FM only males and females.

Table S7: Association between autism features scores and NBS FREE2m in FM only males.

Table S8: Association between corrected intellectual functioning scores and NBS FREE2m in FXS males and females with complete data.

Table S9: Association between autism features scores and NBS FREE2m in FXS males and females with complete data.

Table S10: Association between corrected intellectual functioning scores and DBS FREE2m in FXS males and females.

Table S11: Association between autism features scores and DBS FREE2m in FXS males and females.

Table S12: Association between corrected intellectual functioning scores and DBS FREE2m in FM only males and females.

Table S13: Association between autism features scores and DBS FREE2m in FM only males.

Table S14: Association between corrected intellectual functioning scores and DBS FREE2m in FXS males and females with complete data.

Table S15: Association between autism features scores and DBS FREE2m in FXS males and females with complete data.

Table S16. Spearman's rank correlation (ρ) between FREE2m in NBS and DBS samples with *FMR1* expression in FXS males and females.

Table S17: Spearman's rank correlation (ρ) between NBS and DBS FREE2m and *FMR1* mRNA levels in FM only males and females.

Table S18: Spearman's rank correlations (ρ) between NBS or DBS FREE2m and *FMR1* mRNA levels in FXS males and females with complete data.

Table S1: Correlations between MS-QMA and EpiTYPER in NBS and DBS samples in FXS males.

	MS-QMA*	EpiTYPER#	CPG 1	CpG 2	CpG 6/7	CpG 8/9	CpG 10-12
NBS							
MS-QMA*	32	**0.001+**	**0.003+**	**0.010+**	0.585	**0.001+**	**0.002+**
EpiTYPER#	0.583	30	**<0.001+**	**<0.001+**	**<0.001+**	**<0.001+**	**<0.001+**
CpG 1	0.530	0.644	30	0.061	**0.026+**	**0.007+**	**0.001+**
CpG 2	0.469	0.782	0.353	29	0.140	**0.011+**	**<0.001+**
CpG 6/7	0.104	0.630	0.405	0.281	30	*0.044*	**0.022+**
CpG 8/9	0.566	0.777	0.484	0.467	0.370	30	**<0.001+**
CpG 10-12	0.553	0.878	0.569	0.655	0.416	0.733	30
DBS							
MS-QMA*	24	0.181	*0.043*	0.473	0.584	0.170	*0.039*
EpiTYPER#	0.283	24	**<0.001+**	**<0.001+**	**<0.001+**	**<0.001+**	**<0.001+**
CpG 1	0.416	0.673	24	0.117	**0.005+**	*0.034*	**<0.001+**
CpG 2	0.162	0.886	0.344	22	*0.032*	**0.001+**	**0.001+**
CpG 6/7	-0.121	0.675	0.570	0.468	23	0.187	*0.033*
CpG 8/9	0.296	0.799	0.445	0.692	0.292	23	**0.002+**
CpG 10-12	0.424	0.799	0.745	0.657	0.446	0.621	24

Sample size for each variable (diagonal); Spearman's rank correlation (lower triangle) and its corresponding P-value (upper triangle). *aggregate of CpG's 2-12; #aggregate computed as mean DNAm at CpG sites 1, 2, 6/7, 8/9 and 10-12; FXS = FM only + PM/FM mosaics; DBS = dried blood spot; MS-QMA = methylation specific-quantitative melt analysis; NBS = newborn blood spot; P-values (p) in bold indicate significance for aggregate measures indicating significance; P-values (p) in bold with + remained <0.05 after adjustment for multiple testing for site specific analyses; italicised no longer <0.05 after adjusting for multiple testing for site specific analyses.

Table S2: Correlations between MS-QMA and EpiTYPER in NBS and DBS samples in FXS females.

	MS-QMA*	EpiTYPER#	CPG 1	CpG 2	CpG 6/7	CpG 8/9	CpG 10-12
NBS							
MS-QMA*	20	**0.048**	*0.035*	**0.011+**	0.996	**0.009+**	0.088
EpiTYPER#	0.486	17	**<0.001+**	**0.001+**	**0.002+**	**0.001+**	**0.001+**
CpG 1	0.514	0.913	17	**0.001+**	**0.026+**	**0.001+**	**0.003+**
CpG 2	0.637	0.840	0.799	15	0.256	**0.015+**	*0.043*
CpG 6/7	0.002	0.709	0.553	0.326	16	*0.036*	0.229
CpG 8/9	0.612	0.737	0.715	0.613	0.527	17	**0.001+**
CpG 10-12	0.427	0.738	0.669	0.528	0.319	0.789	17
DBS							
MS-QMA*	18	**0.003+**	**0.006+**	**0.041+**	0.111	**0.005+**	**0.001+**
EpiTYPER#	0.655	18	**0.001+**	**0.007+**	**0.001+**	**0.001+**	**<0.001+**
CpG 1	0.626	0.942	18	**0.026+**	**0.001+**	**0.001+**	**<0.001+**
CpG 2	0.500	0.626	0.538	17	**0.028+**	**0.009+**	**0.020+**
CpG 6/7	0.414	0.887	0.790	0.549	16	**0.001+**	**<0.001+**
CpG 8/9	0.648	0.976	0.939	0.631	0.833	17	**<0.001+**
CpG 10-12	0.732	0.941	0.853	0.576	0.794	0.921	17

Sample size for each variable (diagonal); Spearman's rank correlation (lower triangle) and its corresponding P-value (upper triangle). *aggregate of CpG's 2-12; #aggregate computed as mean DNAm at CpG sites 1, 2, 6/7, 8/9 and 10-12; FXS = FM only + PM/FM mosaics; DBS = dried blood spot; MS-QMA = methylation specific-quantitative melt analysis; NBS = newborn blood spot; P-values (p) in bold indicate significance for aggregate measures indicating significance; P-values (p) in bold with + remained <0.05 after adjustment for multiple testing for site specific analyses; italicised no longer <0.05 after adjusting for multiple testing for site specific analyses.

Table S3: Sensitivity and specificity of NBS FREE2m for FXS versus PM and control females.

			Sensitivity		Specificity		AUC					
	FXS/PM/CN	Threshold	Estimate	95% CI	Estimate	95% CI	Estimate	95% CI	TP	FN	FP	TN
MS-QMA*	21/10/95	0.373	85.7%	63.7%-97.0%	95.2%	89.2%-98.4%	90.5%	82.5%-98.4%	18	3	5	100
EpiTYPER#	19/10/72	0.478	100%	82.4%-100%	98.8%	93.4%-99.9%	99.4%	98.2%-100%	19	0	1	81

The above table presents data from the analyses of EpiTYPER data performed excluding the control outlier (EpiTYPER aggregate MOR = 0.527) and reflects data reported in the paper. Sensitivity, specificity and AUC for FXS versus PMs and controls was estimated using optimal threshold, determined using the Youden method. *aggregate of CpG's 2-12; #aggregate computed as mean DNAm at CpG sites 1, 2, 6/7, 8/9 and 10-12; FXS = FM only + PM/FM mosaics; AUC = area under the receiver operating characteristic (ROC) curve; CI = confidence interval; MS-QMA = methylation specific-quantitative melt analysis; TP = True positive; FN = False negative; FP = False Positive; TN = True negative.

Table S4: Association between each corrected intellectual functioning score and each NBS FREE2m in FXS males and females.

	FXS male				FXS female			
	N	Coef	s.e	p	N	Coef	s.e	p
cFSIQ								
MS–QMA*	24	-58.4	22.1	**0.015**	15	11.7	48.3	0.812
EpiTYPER#	23	-116	23.4	**<0.001**	14	-34.7	61.5	0.583
CpG 1	23	-96.6	32.8	**0.008+**	14	1.59	49.6	0.975
CpG 2	22	-78.0	18.2	**<0.001+**	12	-81.8	60.1	0.203
CpG 6/7	23	-54.2	24.1	**0.036+**	14	-62.7	41.6	0.158
CpG 8/9	23	-82.2	20.3	**0.001+**	14	0.47	48.7	0.993
CpG 10-12	23	-91.1	22.0	**<0.001+**	14	19.2	44.2	0.672
cVIQ								
MS–QMA*	24	-50.5	17.9	**0.010**	15	3.19	34.9	0.927
EpiTYPER#	23	-77.6	21.1	**0.001**	14	-55.3	55.8	0.321
CpG 1	23	-68.0	24.8	**0.012+**	14	-9.75	42.2	0.817
CpG 2	22	-50.4	17.7	**0.010+**	12	-98.7	71.2	0.196
CpG 6/7	23	-38.7	20.7	0.076	14	-74.3	48.1	0.148
CpG 8/9	23	-57.8	18.8	**0.006+**	14	-19.4	41.0	0.637
CpG 10-12	23	-58.6	21.2	**0.012+**	14	9.65	38.9	0.804
cPIQ								
MS–QMA*	24	-81.6	26.2	**0.005**	15	15.6	46.9	0.746
EpiTYPER#	23	-148	28.2	**<0.001**	14	-7.03	62.4	0.912
CpG 1	23	-121	40.7	**0.008+**	14	22.3	52.1	0.677
CpG 2	22	-103	21.1	**<0.001+**	12	-54.6	58.6	0.373
CpG 6/7	23	-65.3	30.1	**0.043+**	14	-26.7	43.9	0.555
CpG 8/9	23	-101	25.5	**<0.001+**	14	8.25	49.5	0.870
CpG 10-12	23	-120	25.8	**<0.001+**	14	11.9	45.6	0.799

Age was controlled in all analyses with FXS male corrected IQ data. Age was not associated with IQ in FXS females and thus was not adjusted. Psychotropic medication use was not associated with IQ results in either sex, thus was not adjusted. *aggregate of CpG's 2-12; #aggregate computed as mean DNAm at CpG sites 1, 2, 6/7, 8/9 and 10-12; FXS = FM only + PM/FM mosaics. cFSIQ = corrected fullscale intelligence quotient; MS-QMA = methylation specific-quantitative melt analysis; NBS = newborn blood spot; cPIQ = corrected performance intelligence quotient; cVIQ = corrected verbal intelligence quotient; +P-value in bold (p) remained <0.05 after adjustment for multiple testing of CpG 1 to CpG10-12.

Table S5: Association between each autism features (ADOS-2 scores) and each NBS FREE2m in FXS males and females.

	FXS male				FXS female			
	N	Coef	s.e	p	N	Coef	s.e	p
ADOS-2 CSS								
MS-QMA*	33	5.96	2.63	**0.030**	17	-4.66	3.67	0.205
EpiTYPER#	31	6.82	4.46	0.137	16	-0.13	14.6	0.993
CpG 1	31	5.26	4.91	0.293	16	0.25	9.16	0.978
CpG 2	30	4.20	2.97	0.169	14	-5.40	4.10	0.188
CpG 6/7	31	-2.05	3.84	0.594	15	8.02	5.43	0.164
CpG 8/9	31	10.1	3.13	**0.003+**	16	0.93	13.3	0.944
CpG 10-12	31	6.69	3.62	0.075	16	-6.53	5.49	0.234
SA CSS								
MS-QMA*	33	5.29	3.06	0.084	17	-2.69	4.19	0.521
EpiTYPER#	31	7.68	10.1	0.447	16	4.07	9.60	0.671
CpG 1	31	6.20	5.16	0.239	16	6.14	8.81	0.485
CpG 2	30	4.12	3.16	0.202	14	-1.52	5.18	0.769
CpG 6/7	31	-2.86	3.77	0.449	15	8.86	6.24	0.179
CpG 8/9	31	8.85	3.25	**0.006+**	16	2.14	9.02	0.813
CpG 10-12	31	7.51	4.88	0.124	16	-2.95	5.14	0.566
RRB CSS								
MS-QMA*	33	2.48	2.09	0.233	17	-4.99	5.28	0.359
EpiTYPER#	31	1.60	3.68	0.665	16	-3.99	7.31	0.594
CpG 1	31	-0.52	3.92	0.896	16	-3.39	5.56	0.552
CpG 2	30	2.00	2.32	0.395	14	0.50	5.27	0.925
CpG 6/7	31	-4.65	2.50	0.063	15	5.80	5.69	0.327
CpG 8/9	31	5.13	2.84	0.071	16	-1.91	6.16	0.761
CpG 10-12	31	1.26	2.51	0.615	16	-4.55	5.66	0.422

As age and medical use were not associated with ADOS-2 in either sex the analyses were not adjusted for these confounders; *aggregate of CpG's 2-12; #aggregate computed as mean DNAm at CpG sites 1, 2, 6/7, 8/9 and 10-12; FXS = FM only + PM/FM mosaics. DBS = dried blood spot; CSS = calibrated severity score; MS-QMA = methylation specific-quantitative melt analysis; NBS = newborn blood spot; RRB = restricted and repetitive behaviours; SA = social affect; +P-value (p) remained <0.05 after adjustment for multiple testing of CpG 1 to CpG10-12.

Table S6: Association between corrected intellectual functioning scores and NBS FREE2m in FM only males and females.

	FM only Males				FM only Females			
	N	Coef	s.e	p	N	Coef	s.e	p
cFSIQ								
MS–QMA*	18	-38.9	9.58	**<0.001**	13	51.2	49.6	0.318
EpiTYPER#	17	-177	28.6	**<0.001**	12	20.2	59.2	0.740
CpG 1	17	-64.8	33.8	0.055	12	29.9	44.6	0.518
CpG 2	16	-68.5	23.0	**0.003+**	10	-53.7	59.1	0.390
CpG 6/7	17	-58.9	21.1	**0.005+**	12	-19.8	43.5	0.660
CpG 8/9	17	-76.3	19.4	**<0.001+**	12	24.7	42.1	0.570
CpG 10-12	17	-139	41.0	**0.001+**	12	47.9	37.0	0.225
cVIQ								
MS–QMA*	18	-51.2	18.9	**0.016**	13	35.8	40.2	0.373
EpiTYPER#	17	-148	17.8	**<0.001**	12	-10.3	61.3	0.867
CpG 1	17	-83.0	27.6	**0.003+**	12	12.8	46.1	0.781
CpG 2	16	-48.9	24.8	*0.049*	10	-81.6	76.9	0.319
CpG 6/7	17	-64.4	15.8	**0.001+**	12	-38.2	56.3	0.513
CpG 8/9	17	-68.8	12.9	**<0.001+**	12	0.09	38.8	0.998
CpG 10-12	17	-97.4	39.6	**0.014+**	12	33.7	36.9	0.359
cPIQ								
MS–QMA*	18	-61.3	32.7	0.081	13	51.2	60.2	0.413
EpiTYPER#	17	-234	33.2	**<0.001**	12	27.1	66.9	0.694
CpG 1	17	-104	73.1	0.176	12	46.8	56.7	0.428
CpG 2	16	-101	28.4	**<0.001+**	10	-40.9	67.5	0.561
CpG 6/7	17	-73.8	29.5	**0.012+**	12	6.71	49.7	0.895
CpG 8/9	17	-96.0	22.4	**<0.001+**	12	24.8	47.9	0.616
CpG 10-12	17	-186	49.8	**<0.001+**	12	24.3	44.8	0.599

Age was controlled in all analyses with FXS male corrected IQ data. Age was not associated with IQ results in FXS females, thus was not adjusted. Psychotropic medication use was not associated with IQ results in FXS males or females, thus was not adjusted. *aggregate of CpG's 2-12; #aggregate computed as mean DNAm at CpG sites 1, 2, 6/7, 8/9 and 10-12; cFSIQ = corrected full scale intelligence quotient; cVIQ = corrected verbal intelligence quotient; cPIQ = corrected performance intelligence quotient; MS-QMA = methylation specific-quantitative melt analysis; NBS = newborn blood spot; P-values (p) in bold indicate significance for aggregate measures indicating significance; P-values (p) in bold with + remained <0.05 after adjustment for multiple testing for site specific analyses; italicised no longer <0.05 after adjusting for multiple testing for site specific analyses.

Table S7: Association between autism features (ADOS-2 scores) and NBS FREE2m in FM only males.

	N	Coef	s.e	p
ADOS-2 CSS				
MS-QMA*	26	4.34	3.03	0.164
EpiTYPER#	24	11.7	7.50	0.134
CpG 1	24	9.17	6.50	0.158
CpG 2	23	1.46	4.34	0.737
CpG 6/7	24	-1.36	4.83	0.778
CpG 8/9	24	11.9	3.66	**0.004+**
CpG 10-12	24	9.41	5.32	0.091
SA CSS				
MS-QMA*	26	3.12	3.87	0.420
EpiTYPER#	24	10.4	8.06	0.210
CpG 1	24	10.9	10.1	0.277
CpG 2	23	2.28	4.83	0.637
CpG 6/7	24	-4.07	5.88	0.488
CpG 8/9	24	8.90	3.57	*0.021*
CpG 10-12	24	9.81	5.63	0.096
RRB CSS				
MS-QMA*	26	1.64	2.46	0.505
EpiTYPER#	24	0.83	6.69	0.902
CpG 1	24	-2.46	5.79	0.671
CpG 2	23	0.82	4.29	0.848
CpG 6/7	24	-7.57	4.77	0.112
CpG 8/9	24	5.53	3.58	0.137
CpG 10-12	24	1.15	4.81	0.813

As age and psychotropic medication use were not associated with ADOS-2 results the analyses were not adjusted for these confounders. *aggregate of CpG's 2-12; #aggregate computed as mean DNAm at CpG sites 1, 2, 6/7, 8/9 and 10-12; DBS = dried blood spot; CSS = calibrated severity score; MS-QMA = methylation specific-quantitative melt analysis; NBS = newborn blood spot; RRB = restricted and repetitive behaviours; SA = social affect; P-values (p) in bold with + remained <0.05 after adjustment for multiple testing for site specific analyses; italicised no longer <0.05 after adjusting for multiple testing for site specific analyses.

Table S8: Association between corrected intellectual functioning scores and NBS FREE2m in FXS males and females with complete data.

	FXS Males				FXS Females			
	N	Coef	s.e	p	N	Coef	s.e	p
cFSIQ								
MS–QMA*	14	-61.8	31.4	**0.049**	12	-20.0	30.2	0.509
EpiTYPER#	13	-96.5	23.2	**0.002**	12	-71.6	46.5	0.124
CpG 1	13	-97.6	32.1	**0.012+**	12	7.70	40.8	0.850
CpG 2	12	-75.1	18.0	**<0.001+**	11	-94.2	46.3	*0.042*
CpG 6/7	12	-47.2	28.4	0.097	11	-60.4	36.5	0.097
CpG 8/9	12	-13.3	27.1	0.635	11	-54.0	41.0	0.188
CpG 10-12	13	-81.2	21.7	**0.004+**	11	-43.3	32.5	0.183
cVIQ								
MS–QMA*	14	-52.0	21.4	**0.015**	12	-37.8	29.7	0.203
EpiTYPER#	13	-56.9	26.0	0.054	12	-101	41.9	**0.016**
CpG 1	13	-56.3	32.1	0.110	12	-36.5	45.1	0.417
CpG 2	12	-45.9	94.2	0.626	11	-134	43.2	**0.002+**
CpG 6/7	12	-38.4	27.3	0.193	11	-67.9	34.5	*0.049*
CpG 8/9	12	6.17	29.2	0.837	11	-87.1	33.0	**0.008+**
CpG 10-12	13	-49.2	23.0	0.058	11	-62.3	29.0	*0.031*
cPIQ								
MS–QMA*	14	-88.0	29.7	**0.003**	12	3.10	32.8	0.924
EpiTYPER#	13	-120	29.0	**0.002**	12	-18.5	47.8	0.698
CpG 1	13	-131	36.9	**0.005+**	12	19.9	55.2	0.719
CpG 2	12	-90.7	25.2	**<0.001+**	11	-46.3	52.6	0.379
CpG 6/7	12	-41.2	23.3	0.077	11	-25.3	41.3	0.540
CpG 8/9	12	-36.7	34.7	0.318	11	-18.2	40.1	0.650
CpG 10-12	13	-103	26.4	**0.003+**	11	-4.10	32.7	0.900

Age was controlled in all analyses; *aggregate of CpG's 2-12; #aggregate computed as mean DNAm at CpG sites 1, 2, 6/7, 8/9 and 10-12; P-values (p) in bold indicate significance for aggregate measures indicating significance; P-values (p) in bold with + remained <0.05 after adjustment for multiple testing for site specific analyses; italicised no longer <0.05 after adjusting for multiple testing for site specific analyses.

Table S9: Association between autism features (ADOS-2 scores) and NBS FREE2m in FXS males and females with complete data.

	FXS Males				FXS Females			
	N	Coef	s.e	p	N	Coef	s.e	p
ADOS-2 CSS								
MS–QMA*	13	0.10	1.22	0.937	12	-0.38	11.2	0.973
EpiTYPER#	12	1.09	1.71	0.536	12	2.92	11.3	0.796
CpG 1	12	1.12	2.03	0.592	12	4.72	8.68	0.587
CpG 2	11	0.88	1.15	0.461	11	4.98	13.6	0.714
CpG 6/7	11	-0.08	1.44	0.954	11	6.57	4.95	0.184
CpG 8/9	11	1.17	2.04	0.579	11	3.55	13.3	0.789
CpG 10-12	12	1.05	1.45	0.486	11	-4.52	11.3	0.688
SA CSS								
MS–QMA*	13	-1.86	2.76	0.515	12	-4.65	8.78	0.596
EpiTYPER#	12	1.65	3.91	0.683	12	4.80	11.2	0.669
CpG 1	12	0.49	4.66	0.918	12	8.69	8.95	0.331
CpG 2	11	0.59	3.09	0.852	11	2.56	12.7	0.840
CpG 6/7	11	0.34	3.12	0.915	11	7.34	3.66	*0.045*
CpG 8/9	11	7.02	3.84	0.100	11	4.44	12.2	0.715
CpG 10-12	12	1.46	3.34	0.672	11	-2.27	10.2	0.825
RRB CSS								
MS–QMA*	14	2.38	2.29	0.298	12	7.28	4.12	0.077
EpiTYPER#	13	-1.32	3.88	0.733	12	4.26	6.19	0.491
CpG 1	13	0.56	3.31	0.867	12	1.41	5.53	0.798
CpG 2	12	1.44	2.79	0.605	11	15.3	7.55	*0.043*
CpG 6/7	12	-4.34	2.45	0.076	11	4.05	6.52	0.534
CpG 8/9	12	-4.25	4.64	0.359	11	4.35	4.46	0.330
CpG 10-12	13	-0.46	2.59	0.859	11	2.17	5.73	0.705

Age was not associated with autism features scores, in both males and females in this sample and therefore not adjusted for; *aggregate of CpG's 2-12; #aggregate computed as mean DNAm at CpG sites 1, 2, 6/7, 8/9 and 10-12. P-values (p) italicised no longer <0.05 after adjusting for multiple testing for site specific analyses.

Table S10: Association between corrected intellectual functioning scores and DBS FREE2m in FXS males and females.

	FXS males				FXS females			
	N	Coef	s.e	p	N	Coef	s.e	p
cFSIQ								
MS-QMA*	19	-0.29	22.6	0.990	14	51.9	49.6	0.318
EpiTYPER#	19	-35.9	46.3	0.438	14	20.2	59.2	0.740
CpG 1	19	-17.8	40.9	0.664	14	29.9	44.6	0.518
CpG 2	18	-29.6	28.4	0.298	14	-53.7	59.1	0.390
CpG 6/7	18	-18.4	27.7	0.508	13	-19.8	43.5	0.660
CpG 8/9	18	2.82	24.2	0.907	13	24.7	42.1	0.570
CpG 10-12	19	-33.2	36.8	0.367	13	47.9	37.0	0.225
cVIQ								
MS-QMA*	19	-6.14	18.1	0.740	14	80.8	92.5	0.382
EpiTYPER#	19	-35.2	18.4	0.074	14	-40.8	48.8	0.403
CpG 1	19	-22.0	22.4	0.325	14	-10.1	22.5	0.654
CpG 2	18	-25.7	12.1	0.051	14	-50.1	118	0.671
CpG 6/7	18	-17.7	17.5	0.329	13	-64.0	33.3	0.055
CpG 8/9	18	-16.2	22.7	0.485	13	-50.1	47.9	0.295
CpG 10-12	19	-38.7	19.7	0.067	13	-40.7	41.7	0.329
cPIQ								
MS-QMA*	19	-40.1	25.8	0.139	14	77.8	111	0.496
EpiTYPER#	19	-46.9	59.1	0.427	14	21.7	51.1	0.679
CpG 1	19	-16.2	37.7	0.668	14	21.6	37.5	0.575
CpG 2	18	-49.3	17.1	*0.011*	14	28.0	67.0	0.684
CpG 6/7	18	-19.1	28.2	0.497	13	-10.4	44.7	0.821
CpG 8/9	18	-8.60	29.3	0.773	13	25.6	56.4	0.659
CpG 10-12	19	-32.9	50.8	0.518	13	14.8	41.9	0.730

Age was controlled in all analyses with FXS male IQ data. Age was not associated with IQ results in FXS females, so the analysis was not adjusted for this variable. Psychotropic medication use was not associated with IQ results in FXS males or females, thus was not adjusted for in the analyses. *aggregate of CpG's 2-12; #aggregate computed as mean DNAm at CpG sites 1, 2, 6/7, 8/9 and 10-12; FXS = FM only + PM/FM mosaics; DBS = dried blood spot; cFSIQ = corrected full scale intelligence quotient; MS-QMA = methylation specific-quantitative melt analysis; cPIQ = corrected performance intelligence quotient; cVIQ = corrected verbal intelligence quotient. P-values (p) italicised no longer <0.05 after adjusting for multiple testing for site specific analyses.

Table S11: Association between autism features (ADOS-2 scores) and DBS FREE2m in FXS males and females.

	FXS males				FXS females			
	N	Coef	s.e	p	N	Coef	s.e	p
ADOS-2 CSS								
MS-QMA*	24	5.01	3.15	0.126	17	-13.4	9.87	0.196
EpiTYPER#	24	-0.68	3.55	0.849	17	-0.43	5.11	0.934
CpG 1	24	0.85	3.49	0.811	17	0.75	3.51	0.833
CpG 2	22	-0.73	2.36	0.762	16	-5.57	7.33	0.461
CpG 6/7	23	-3.66	2.93	0.224	15	3.37	3.84	0.396
CpG 8/9	23	3.43	4.08	0.410	16	0.51	4.12	0.902
CpG 10-12	24	0.19	3.85	0.962	16	-0.89	4.55	0.847
SA CSS								
MS-QMA*	24	4.01	3.40	0.251	17	-11.7	10.3	0.275
EpiTYPER#	24	0.66	3.74	0.862	17	1.13	5.27	0.833
CpG 1	24	1.24	3.68	0.738	17	2.16	3.58	0.556
CpG 2	22	0.26	2.49	0.919	16	-5.76	7.59	0.460
CpG 6/7	23	-2.07	3.16	0.520	15	5.21	3.79	0.192
CpG 8/9	23	4.86	4.23	0.264	16	2.00	4.18	0.640
CpG 10-12	24	1.68	4.04	0.682	16	0.30	4.80	0.951
RRB CSS								
MS-QMA*	24	2.61	2.81	0.362	17	-3.91	7.38	0.604
EpiTYPER#	24	-4.42	2.91	0.143	17	-1.01	3.64	0.785
CpG 1	24	-4.53	2.85	0.127	17	-0.28	2.50	0.912
CpG 2	22	-1.50	1.73	0.386	16	0.37	5.25	0.945
CpG 6/7	23	-5.00	2.07	*0.016*	15	-1.17	3.08	0.710
CpG 8/9	23	-2.45	3.53	0.495	16	0.12	3.28	0.971
CpG 10-12	24	-4.73	3.16	0.148	16	-1.27	3.18	0.695

As age and psychotropic medication use were not associated with ADOS-2 results in FXS males and females, the analysis was not adjusted for these confounders. *aggregate of CpG's 2-12; #aggregate computed as mean DNAm at CpG sites 1, 2, 6/7, 8/9 and 10-12; FXS = FM only + PM/FM mosaics. ADOS = Autism diagnostic observation scale; CSS = calibrated severity score; DBS = dried blood spot; MS-QMA = methylation specific-quantitative melt analysis; RRB = restricted and repetitive behaviours; SA = social affect. P-values (p) italicised no longer <0.05 after adjusting for multiple testing for site specific analyses.

Table S12: Association between corrected intellectual functioning scores and DBS FREE2m in FM only males and females.

	FM only males				FM only females			
	N	Coef	s.e	p	N	Coef	s.e	p
cFSIQ								
MS-QMA*	15	15.2	17.2	0.377	12	141	90.1	0.148
EpiTYPER#	15	3.46	18.5	0.852	12	35.2	45.1	0.453
CpG 1	15	21.8	14.4	0.129	12	21.6	29.7	0.483
CpG 2	14	-6.37	19.7	0.747	12	56.6	61.7	0.380
CpG 6/7	14	10.4	12.0	0.385	11	-7.79	41.9	0.857
CpG 8/9	15	2.29	28.6	0.937	11	34.6	48.6	0.495
CpG 10-12	15	-7.74	20.4	0.705	11	33.7	37.4	0.392
cVIQ								
MS-QMA*	15	14.3	11.7	0.223	12	104	86.9	0.233
EpiTYPER#	15	-33.2	26.4	0.233	12	-8.63	37.9	0.820
CpG 1	15	-2.38	23.7	0.920	12	-2.82	22.8	0.902
CpG 2	14	-23.7	12.8	0.090	12	2.71	59.0	0.963
CpG 6/7	14	-8.71	17.4	0.616	11	-40.3	36.9	0.275
CpG 8/9	15	-31.8	24.3	0.215	11	-16.6	38.4	0.666
CpG 10-12	15	-37.0	25.5	0.172	11	-7.02	29.0	0.808
cPIQ								
MS-QMA*	15	-1.46	24.8	0.953	12	93.3	121	0.457
EpiTYPER#	15	0.73	27.0	0.978	12	41.0	53.8	0.463
CpG 1	15	17.0	13.2	0.197	12	22.9	34.5	0.523
CpG 2	14	-28.5	29.6	0.335	12	66.9	66.7	0.339
CpG 6/7	14	4.46	20.1	0.824	11	9.11	54.7	0.871
CpG 8/9	15	-10.4	35.4	0.774	11	46.6	56.5	0.431
CpG 10-12	15	9.22	22.2	0.678	11	48.3	44.2	0.303

Age was controlled in all analyses with FXS male IQ data. Age was not associated with IQ results in FXS females, so the analysis was not adjusted for this variable. Psychotropic medication use was not associated with IQ results in FXS males or females, thus was not adjusted for in the analyses. *aggregate of CpG's 2-12; #aggregate computed as mean DNAm at CpG sites 1, 2, 6/7, 8/9 and 10-12; DBS = dried blood spot; MS-QMA = methylation specific-quantitative melt analysis; cFSIQ = corrected full scale intelligence quotient; cPIQ = corrected performance intelligence quotient; cVIQ = corrected verbal intelligence quotient.

Table S13: Association between autism features (ADOS-2 scores) and DBS FREE2m in FM only males.

	FM only males			
	N	Coef	s.e	p
ADOS-2 CSS				
MS-QMA*	20	4.13	3.74	0.284
EpiTYPER#	20	-0.86	3.08	0.781
CpG 1	20	0.52	4.35	0.907
CpG 2	18	0.29	1.65	0.862
CpG 6/7	19	-4.01	3.69	0.278
CpG 8/9	20	3.40	4.24	0.432
CpG 10-12	20	-1.24	4.83	0.800
SA CSS				
MS-QMA*	20	3.18	3.99	0.435
EpiTYPER#	20	0.35	5.66	0.950
CpG 1	20	1.43	4.55	0.756
CpG 2	18	0.78	3.51	0.824
CpG 6/7	19	-2.12	4.58	0.643
CpG 8/9	20	3.30	4.46	0.469
CpG 10-12	20	1.10	5.07	0.831
RRB CSS				
MS-QMA*	20	2.45	3.52	0.495
EpiTYPER#	20	-7.62	4.12	0.081
CpG 1	20	-7.01	3.67	0.072
CpG 2	18	-1.69	2.53	0.505
CpG 6/7	19	-7.25	3.12	0.033
CpG 8/9	20	-2.37	3.95	0.556
CpG 10-12	20	-7.71	4.09	0.075

As age and medical use were not associated with ADOS-2 results in FM only males, the analysis was not adjusted for these confounders. *aggregate of CpG's 2-12; #aggregate computed as mean DNAm at CpG sites 1, 2, 6/7, 8/9 and 10-12; ADOS = Autism diagnostic observation scale; CSS = calibrated severity score; DBS = dried blood spot; MS-QMA = methylation specific-quantitative melt analysis; RRB = restricted and repetitive behaviours; SA = social affect.

Table S14: Association between corrected intellectual functioning scores and DBS FREE2m in FXS males and females with complete data.

	FXS Males				FXS Females			
	N	Coef	s.e	p	N	Coef	s.e	p
cFSIQ								
MS–QMA*	14	-16.4	42.9	0.702	12	9.24	68.8	0.896
EpiTYPER#	13	-68.1	21.9	**0.011**	12	-41.0	38.9	0.292
CpG 1	13	-42.6	55.7	0.444	12	-10.1	23.8	0.671
CpG 2	12	-50.0	16.3	*0.014*	11	-6.50	57.1	0.910
CpG 6/7	12	-35.7	38.5	0.354	11	-49.7	30.0	0.137
CpG 8/9	12	-23.7	25.2	0.370	11	-46.8	49.5	0.372
CpG 10-12	13	-73.2	29.3	*0.032*	11	-32.6	33.5	0.332
cVIQ								
MS–QMA*	14	-3.30	23.2	0.887	12	10.6	92.7	0.911
EpiTYPER#	13	-49.4	20.0	**0.033**	12	-66.6	36.1	0.065
CpG 1	13	-41.5	28.0	0.139	12	-27.9	23.0	0.225
CpG 2	12	-34.7	14.2	*0.036*	11	-40.1	45.6	0.379
CpG 6/7	12	-37.0	23.3	0.113	11	-59.2	30.1	0.084
CpG 8/9	12	-21.4	27.2	0.451	11	-81.2	46.8	0.121
CpG 10-12	13	-52.2	21.7	*0.037*	11	-50.5	36.3	0.165
cPIQ								
MS–QMA*	14	-39.2	51.1	0.443	12	29.7	74.7	0.700
EpiTYPER#	13	-86.3	31.2	**0.020**	12	7.00	47.1	0.882
CpG 1	13	-35.6	59.1	0.547	12	11.8	24.0	0.623
CpG 2	12	-70.1	17.2	**0.003+**	11	24.1	35.3	0.494
CpG 6/7	12	-29.6	35.1	0.399	11	-11.5	34.4	0.747
CpG 8/9	12	-45.7	32.0	0.187	11	8.42	51.9	0.875
CpG 10-12	13	-85.7	30.9	**0.020+**	11	5.60	34.1	0.869

Age was controlled in all analyses; *aggregate of CpG's 2-12; #aggregate computed as mean DNAm at CpG sites 1, 2, 6/7, 8/9 and 10-12; P-values (p) in bold indicate significance for aggregate measures indicating significance; P-values (p) in bold with + remained <0.05 after adjustment for multiple testing for site specific analyses; italicised no longer <0.05 after adjusting for multiple testing for site specific analyses

Table S15: Association between autism features (ADOS-2 scores) and DBS FREE2m in FXS males and females with complete data.

	FXS Males				FXS Females			
	N	Coef	s.e	p	N	Coef	s.e	p
ADOS-2 CSS								
MS–QMA*	13	0.31	1.13	0.789	12	-17.5	13.0	0.180
EpiTYPER#	12	-0.61	1.40	0.672	12	2.66	6.64	0.689
CpG 1	12	-0.93	1.27	0.481	12	1.56	3.45	0.652
CpG 2	11	0.51	0.90	0.583	11	-9.01	6.85	0.188
CpG 6/7	11	-0.19	1.20	0.876	11	5.22	4.19	0.213
CpG 8/9	11	-1.62	1.64	0.349	11	4.71	8.01	0.556
CpG 10-12	12	-1.62	1.48	0.300	11	3.28	5.69	0.564
SA CSS								
MS–QMA*	13	-0.74	2.61	0.782	12	-12.1	14.2	0.393
EpiTYPER#	12	-1.76	3.16	0.589	12	4.52	6.14	0.462
CpG 1	12	-1.45	2.90	0.629	12	3.95	3.13	0.207
CpG 2	11	0.08	2.40	0.974	11	-5.50	8.58	0.521
CpG 6/7	11	-0.62	2.60	0.818	11	6.15	3.66	0.093
CpG 8/9	11	-2.37	3.66	0.534	11	6.35	6.99	0.363
CpG 10-12	12	-2.65	3.45	0.459	11	3.93	4.95	0.427
RRB CSS								
MS–QMA*	14	1.48	1.71	0.385	12	-13.7	10.0	0.174
EpiTYPER#	13	-2.08	3.80	0.585	12	-0.26	5.39	0.962
CpG 1	13	-3.67	2.66	0.168	12	-1.72	2.38	0.470
CpG 2	12	0.39	2.09	0.852	11	4.37	11.3	0.698
CpG 6/7	12	-3.69	2.36	0.117	11	3.57	3.10	0.249
CpG 8/9	12	-0.92	3.80	0.809	11	0.24	5.32	0.964
CpG 10-12	13	-1.09	3.09	0.724	11	2.61	4.88	0.592

Age was not associated with autism features scores in both males and females in this sample and therefore was not adjusted for; *aggregate of CpG's 2-12; #aggregate computed as mean DNAm at CpG sites 1, 2, 6/7, 8/9 and 10-12.

Table S16. Spearman's rank correlation (ρ) between FREE2m in NBS and DBS samples with *FMR1* expression in both sexes.

	FXS male			FXS female		
	N	$\rho \pm$ SE	p	N	$\rho \pm$ SE	p
NBS						
MS-QMA*	13	-0.30 ± 0.29	0.316	15	-0.23 ± 0.27	0.419
EpiTYPER#	13	-0.58 ± 0.25	**0.037**	14	-0.65 ± 0.22	**0.012**
CpG 1	13	-0.63 ± 0.23	0.022	14	-0.54 ± 0.24	0.046
CpG 2	13	-0.50 ± 0.26	0.082	13	-0.20 ± 0.30	0.505
CpG 6/7	13	-0.38 ± 0.28	0.201	13	-0.60 ± 0.24	**0.029+**
CpG 8/9	13	-0.68 ± 0.27	**0.009+**	14	-0.75 ± 0.19	**0.002+**
CpG 10-12	13	-0.46 ± 0.25	0.117	14	-0.84 ± 0.16	**<0.001+**
DBS						
MS-QMA*	17	-0.27 ± 0.25	0.291	16	-0.69 ± 0.19	**0.003**
EpiTYPER#	17	-0.66 ± 0.19	**0.004**	16	-0.82 ± 0.15	**<0.001**
CpG 1	17	-0.42 ± 0.23	0.090	16	-0.76 ± 0.17	**<0.001+**
CpG 2	17	-0.64 ± 0.20	**0.005+**	15	-0.35 ± 0.26	0.205
CpG 6/7	16	-0.50 ± 0.23	0.048	14	-0.87 ± 0.14	**<0.001+**
CpG 8/9	16	-0.38 ± 0.25	0.147	15	-0.78 ± 0.17	**<0.001+**
CpG 10-12	17	-0.50 ± 0.22	0.042	15	-0.80 ± 0.17	**<0.001+**

Male results were first log transformed with cases with zero *FMR1* mRNA removed from the analysis. Female data was not transformed. *aggregate of CpG's 2-12; #aggregate computed as mean DNAm at CpG sites 1, 2, 6/7, 8/9 and 10-12). DBS = dried blood spot; MS-QMA = methylation specific-quantitative melt analysis; NBS = newborn blood spot; +P-value in bold (p) remained <0.05 after adjustment for multiple testing of CpG 1 to CpG10-12.

Table S17: Spearman's rank correlation (ρ) between NBS and DBS FREE2m and *FMR1* mRNA levels in FM only males and females.

	FM only males			FM only females		
	N	ρ	p	N	ρ	p
NBS						
MS-QMA*	10	-0.152	0.676	14	-0.209	0.473
EpiTYPER#	10	-0.091	0.803	13	-0.654	**0.015**
CpG 1	10	-0.176	0.626	13	-0.603	**0.029+**
CpG 2	10	-0.091	0.803	12	-0.266	0.403
CpG 6/7	10	0.333	0.347	12	-0.525	0.080
CpG 8/9	10	-0.310	0.383	13	-0.747	**0.003+**
CpG 10-12	10	0.103	0.777	13	-0.845	**<0.001+**
DBS						
MS-QMA*	13	-0.220	0.471	15	-0.661	**0.007**
EpiTYPER#	13	-0.388	0.190	15	-0.825	**<0.001**
CpG 1	13	-0.126	0.681	15	-0.800	**<0.001+**
CpG 2	13	-0.465	0.109	14	-0.196	0.501
CpG 6/7	12	-0.004	0.991	13	-0.868	**<0.001+**
CpG 8/9	13	-0.308	0.306	14	-0.780	**0.001+**
CpG 10-12	13	-0.291	0.334	14	-0.763	**0.002+**

Male results were first log transformed with cases with zero *FMR1* mRNA removed from the analysis. Female data was not transformed. *aggregate of CpG's 2-12; #aggregate computed as mean DNAm at CpG sites 1, 2, 6/7, 8/9 and 10-12. DBS = dried blood spot; MS-QMA = methylation specific-quantitative melt analysis; NBS = newborn blood spot; P-values (p) in bold indicate significance for aggregate measures indicating significance; P-values (p) in bold with + remained <0.05 after adjustment for multiple testing for site specific analyse.

Table S18: Spearman's rank correlations (*ρ*) between NBS or DBS FREE2m and *FMR1* mRNA levels in FXS males and females with complete data.

	FXS males			FXS females		
	N	*ρ*	p	N	*ρ*	p
NBS						
MS-QMA*	12	-0.10	0.762	12	-0.18	0.586
EpiTYPER#	11	-0.48	0.133	12	-0.51	0.090
CpG 1	11	-0.66	*0.026*	12	-0.50	0.101
CpG 2	11	-0.53	0.096	11	-0.17	0.610
CpG 6/7	10	-0.62	0.054	11	-0.54	0.089
CpG 8/9	10	-0.16	0.663	11	-0.64	*0.035*
CpG 10-12	11	-0.32	0.340	11	-0.59	0.056
DBS						
MS-QMA*	12	-0.21	0.512	12	-0.67	**0.018**
EpiTYPER#	11	-0.62	**0.040**	12	-0.74	**0.006**
CpG 1	11	-0.50	0.117	12	-0.76	**0.004+**
CpG 2	11	-0.64	*0.033*	11	-0.26	0.432
CpG 6/7	10	-0.55	0.098	11	-0.65	**0.029+**
CpG 8/9	10	-0.54	0.111	11	-0.77	**0.005+**
CpG 10-12	11	-0.85	**0.001+**	11	-0.74	**0.010+**

Male results were first log transformed with cases with zero *FMR1* mRNA removed from the analysis. Female data was not transformed. *Aggregate of CpG's 2-12; #aggregate computed as mean DNAm at CpG sites 1, 2, 6/7, 8/9 and 10-12; P-value in bold is significant; P-values (p) in bold indicate significance for aggregate measures indicating significance; P-values (p) in bold with + remained <0.05 after adjustment for multiple testing for site specific analyses; italicised no longer <0.05 after adjusting for multiple testing for site specific analyses
